# First Reported Use of aiva® Re-Verse, a Novel Combination Injection Protocol for Facial Rejuvenation: A Report of Three Cases

**DOI:** 10.7759/cureus.89664

**Published:** 2025-08-09

**Authors:** Mohammed Chaudhry

**Affiliations:** 1 Aesthetics, Aiva Clinic, London, GBR

**Keywords:** calcium hydroxyapatite, dermal filler, facelift, facial rejuvenation, filler, hyaluronic acid, pdrn

## Abstract

Facial rejuvenation (FR) remains one of the most sought-after treatments in aesthetic medicine. This case series demonstrates FR using aiva® Re-Verse (Aiva Services Ltd., London, United Kingdom), a proprietary formulation combining hyper-dilute Radiesse® (calcium hydroxylapatite (CaHA); Merz Pharmaceuticals GmbH, Frankfurt, Germany), Plinest® (polydeoxyribonucleotide (PDRN); Mastelli Srl, Italy), and Profhilo® (stabilised hybrid cooperative complex of high- and low-molecular-weight hyaluronic acid (HA) 64 mg per 2 ml; IBSA Farmaceutici Italia, Lodi, Italy).

Unlike traditional protocols where these compounds are administered individually and typically on different treatment dates, aiva® Re-Verse allows all three components to be reconstituted together via an aseptic closed Luer-to-Luer transfer system. The pre-mixed compound, which contains all three ingredients, was delivered simultaneously within each injection bolus in one treatment session.

To date, no published data have reported on the efficacy of this trifecta administered as a unified formulation. Three female subjects aged 44, 57, and 59 (mean age: 53.3 years) underwent FR with aiva® Re-Verse. Outcomes were assessed six months later by the subjects and treating doctor using the Global Aesthetic Improvement Scale (GAIS). All cases showed a maximum improvement post-treatment on the GAIS, indicating a successful response to intervention. No adverse events, such as nodules, hypersensitivity reactions, and/or delayed complications, were observed. Treatment was well tolerated, producing global aesthetic improvements, including enhanced hydration, subtle volumisation, improved elasticity, and refined skin texture.

This technique offers a streamlined, cost-effective, and minimally invasive alternative to multi-step rejuvenation procedures. The limitations of non-cross-linked HA in terms of diffusion and degradation suggest that future studies should explore mildly cross-linked alternatives in combination with PDRN and CaHA, which may offer improved tissue re-density, hydration retention, and overall clinical outcomes. These promising results warrant further investigation in larger cohorts with extended follow-up.

## Introduction

Facial ageing is a multifactorial process characterised by volume loss, dermal thinning, loss of elasticity, and deteriorating skin quality [1]. While traditional rejuvenation treatments have primarily focused on volumising agents to address structural deficits, the field of regenerative aesthetic medicine has increasingly shifted toward therapies that stimulate the skin's intrinsic repair mechanisms [2-8]. This paradigm shift reflects a growing recognition that rejuvenation requires both immediate correction and long-term dermal remodelling.

Conventionally, bio-stimulatory and hydrating compounds, such as calcium hydroxylapatite (CaHA), polydeoxyribonucleotide (PDRN), and hyaluronic acid (HA), are administered individually, either in separate anatomical layers or across multiple treatment sessions on different dates [2-5]. While clinically effective, this segmented approach is often associated with higher patient costs, increased patient discomfort, and reduced adherence to treatment protocols due to the repetitive nature of sessions [2-5].

aiva® Re-Verse (Aiva Services Ltd., London, United Kingdom) was developed to overcome these limitations by integrating three compounds, namely, hyper-dilute Radiesse® (CaHA; Merz Pharmaceuticals GmbH, Frankfurt, Germany), Plinest® (PDRN; Mastelli Srl, Italy), and Profhilo® (stabilised hybrid cooperative complex of high- and low-molecular-weight HA; IBSA Farmaceutici Italia, Lodi, Italy), into a unified single injectable formulation. This novel approach allows all three agents to be delivered simultaneously within each single injection bolus, optimising procedural efficiency and patient experience. The combined formulation is designed to deliver both immediate and progressive outcomes: HA provides instant hydration, subtle volumisation, and lifting effects, while CaHA and PDRN synergistically stimulate neocollagenesis [2-5]. The compounds together create a biological scaffold that supports fibroblast activity, promoting the deposition of new collagen fibres and sustained dermal revitalisation [2-5]. 

This case series explores the application of aiva® Re-Verse in three female subjects, highlighting its potential to streamline treatment while enhancing outcomes and reducing overall treatment burden.

## Case presentation

Three female subjects (aged 44, 57, and 59) presented with concerns regarding early facial ageing, including mild volume loss, decreased skin elasticity, and dull complexion. All subjects were previously fit and well, with no comorbidities; they all maintained healthy lifestyle habits and were not on medications affecting dermal quality.

Injection protocol

The aiva® Re-Verse formulation was prepared under aseptic conditions by reconstituting the following quantities together using a closed Luer-to-Luer transfer system (Rapid Fill Luer-to-Luer Connector, Baxter Healthcare Ltd., Deerfield, Illinois, United States). The following were reconstituted together: 1.5 ml CaHA, 2 ml 0.9% normal saline, 0.5 ml 2% lidocaine, 1 ml 2% PDRN solution, and 1 ml of 32 mg/ml non-cross-linked HA.

All compounds were placed into one sterile 10 ml syringe (BD Plastipak™, Becton Dickinson, Franklin Lakes, New Jersey, United States) via the Luer-to-Luer connector. Once all compounds were placed into one 10 ml syringe, an empty sterile 10 ml syringe was attached to the other side of the Luer-to-Luer connector. To ensure adequate mixing and molecular dispersion, the compounds were passed back and forth between the syringes exactly 10 times, applying consistent manual pressure to facilitate turbulent flow and shear homogenisation. The final mixture demonstrated complete macroscopic homogeneity, with no visible phase separation, air bubbles, or particulate matter. The preparation was immediately transferred into a sterile labelled delivery syringe for clinical use. The final formulation yielded 6 ml of pre-mixed, sterile injectable solution, ready for application. The pre-mixed sterile compound was aseptically decanted via a closed sterile Luer-to-Luer transfer connector into 1 ml BD Plastipak™ Luer-Lock syringes (Becton Dickinson, Franklin Lakes, New Jersey, United States). Prior to administration, a 27-gauge, 13 mm grey hypodermic needle (BD Microlance™, Becton Dickinson, Franklin Lakes, New Jersey, United States) was affixed to each syringe to facilitate precise dermal injection.

Injection technique

**Figure 1:**
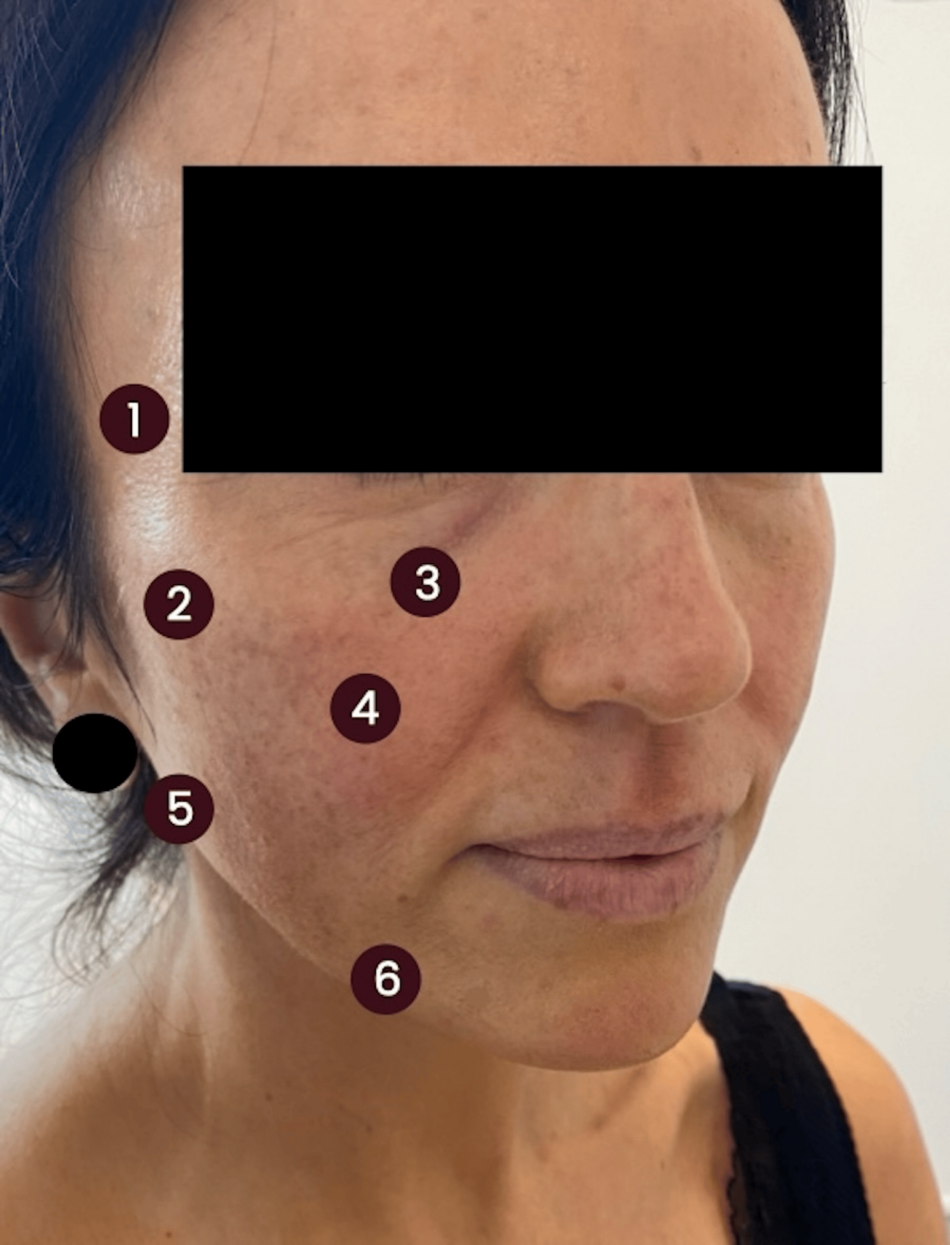
An annotated frontal-oblique angle clinical photograph Numbers 1-6 highlight the injection points for each side of the face.

The injection protocol utilised a 6-point technique per side (Figure 1). Exactly 0.5 ml was administered at each point. A total of 3 ml was administered to each side of the face. 

**Figure 2:**
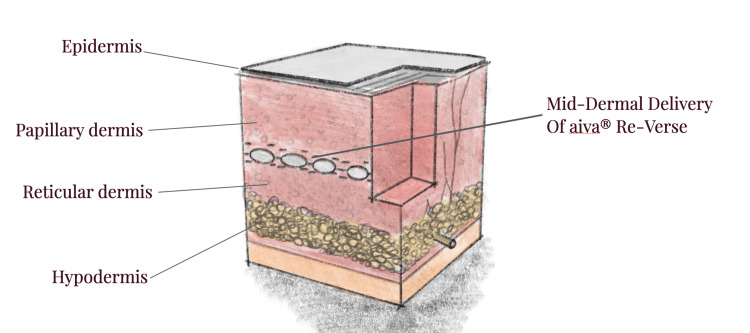
Schematic cross section of the facial skin The schema highlights the target injection depth (mid-dermis) of the compounds at each injection point. Image Credit: Mohammed Chaudhry

The injection depth was mid-dermis (Figure 2).

Immediately following injection, sterile gauze soaked in chlorhexidine gluconate was applied with firm pressure to each bolus site to encourage even dispersion and integration of the product. Meticulous care was taken to avoid inadvertent intravascular delivery; capillary refill was assessed, and aspiration was performed prior to each injection to ensure vascular safety and confirm intact perfusion.

Outcome measures

Six months post-procedurally, the patients and treating doctor completed the evaluation using the Global Aesthetic Improvement Scale (GAIS), a well-known validated tool used in aesthetic medicine for measuring satisfaction with aesthetic outcomes (Table 1). A successful outcome was defined as an improvement of at least 1 point on the GAIS, indicating a visible and satisfactory enhancement in facial appearance.

**Table 1:** Global Aesthetic Improvement Scale

Score	Grade
3	Very much improved in appearance
2	Much improved in appearance
1	Improved in appearance
0	No change in appearance
-1	Appearance worsened after treatment
-2	Appearance very much worsened after treatment

Results

Case 1

**Figure 3:**
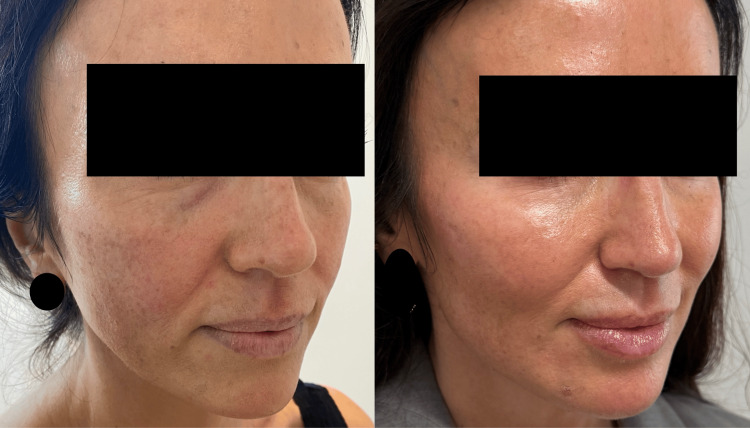
Case 1: side-by-side comparison of before (A) and six months after the treatment (B) Note the improved fullness in the mid-cheek, reduction in tear-trough hollowing, and improved skin texture and brightness. There is also a subtle lift of jowls along the jawline.

This 44-year-old woman presented with early-onset changes of ageing in the face. She noticed an increase in volume loss in her mid-face. The volume loss has led to the ptosis of mid-face compartments and laxity of retaining facial ligaments, leading to a worse appearance of her tear-trough region, as well as a thinning of the skin. She underwent treatment of aiva® Re-Verse as described. Her GAIS score, according to the author and the patient, at six months post-treatment was 3 (Figure 3). 

Case 2

**Figure 4:**
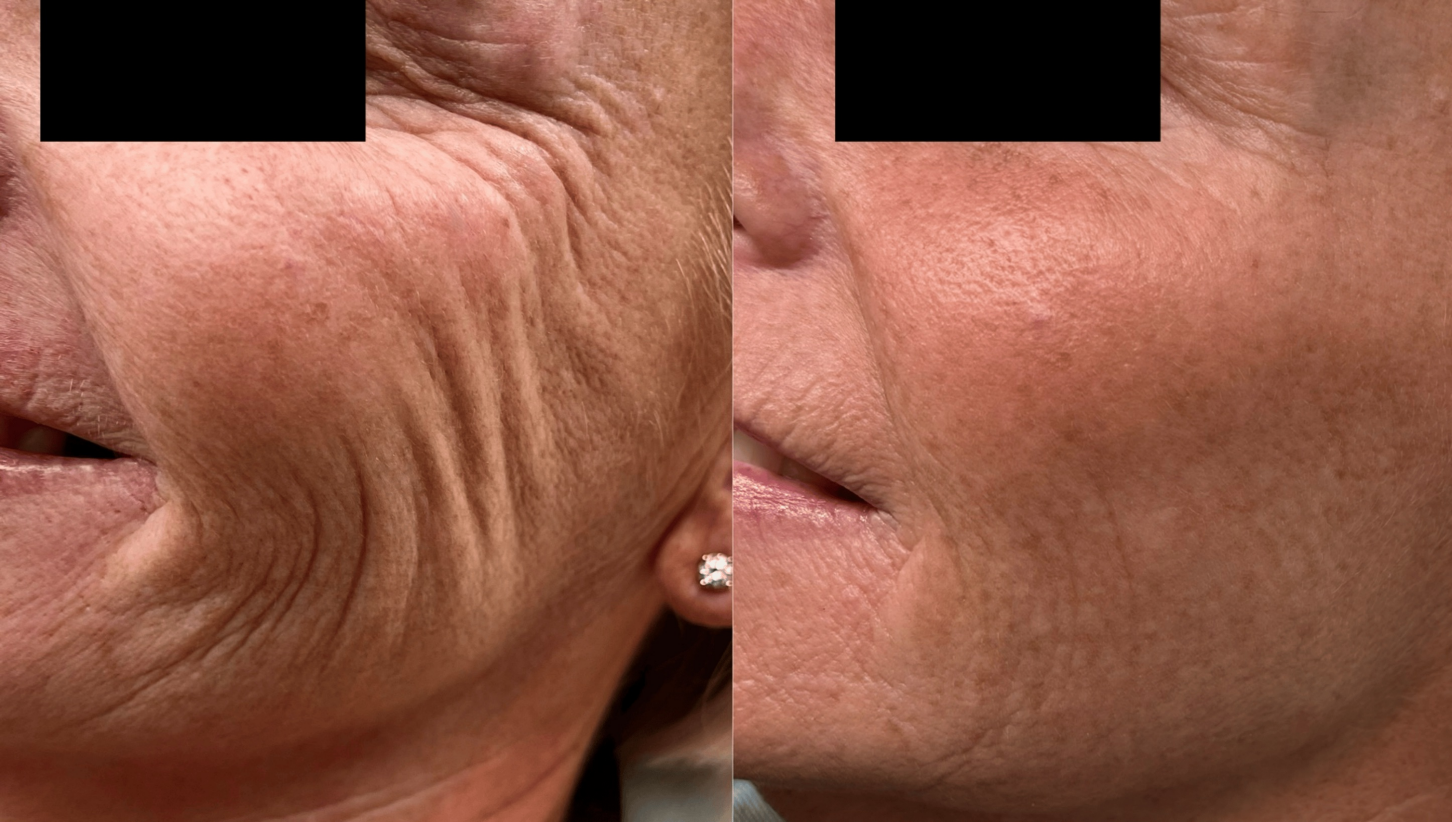
Case 2: side-by-side comparison of before (A) and six months after the treatment (B) The subject was asked to smile in clinical photograph (A) and (B) to demonstrate dynamic as well as static lines. Note the reduction in static and dynamic lines as well as improvement in skin texture and brightness.

**Figure 5:**
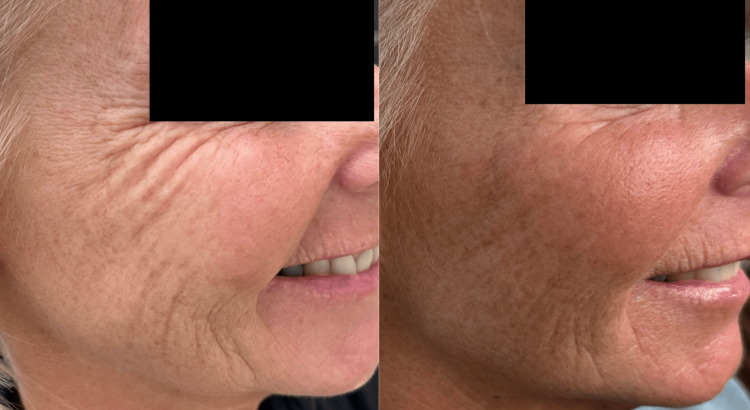
Case 2: side-by-side comparison of before (A) and six months after the treatment (B) The subject was asked to smile in clinical photograph (A) and (B) to demonstrate dynamic as well as static lines. Note the reduction in static and dynamic lines around the eyes, lateral zygoma, and peri-oral area.

This 57-year-old woman presented with mid-late-onset changes of ageing in the face. She was particularly concerned by the increase in static and dynamic lines (upon smiling and expression) around her eyes, mid-lateral cheeks, and jawline. She underwent treatment of aiva® Re-Verse as described. Her GAIS score, according to the author and the patient, at six months post-treatment was 3, indicating a significant improvement in her appearance (Figures 4-5).

Case 3

**Figure 6:**
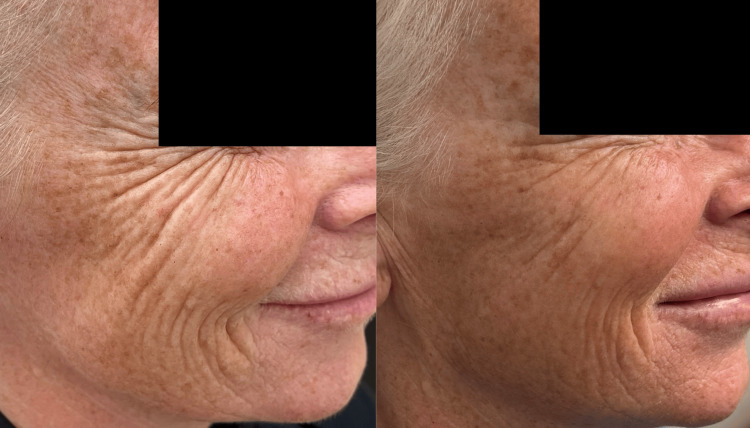
Case 3: side-by-side comparison of before (A) and six months after the treatment (B) The subject was asked to smile in clinical photograph (A) and (B) to demonstrate dynamic as well as static lines. Note the reduction in lines peri-orbitally and peri-orally as well as moderate improvement in skin texture and brightness.

This 59-year-old woman presented with late-onset changes of ageing in the face. She was particularly concerned by the increase in deep static and dynamic lines (upon smiling and expression) around her eyes, mid-lateral cheeks, jawline, and mouth. She underwent treatment of aiva® Re-Verse as described. Her GAIS score, according to the author and the patient, at six months post-treatment was 3, indicating a significant improvement in her appearance (Figure 6).

## Discussion

The aiva® Re-Verse protocol is predicated on the simultaneous intradermal delivery of the three bioactive compounds previously discussed, with each one targeting a distinct cellular pathway involved in dermal repair, hydration, and neocollagenesis [2-8]. Unlike conventional monotherapy approaches, which administer these single agents in isolation, this protocol combines all three agents, which are known to stimulate collagen production through complementary mechanisms, such as fibroblast activation, angiogenesis, and extracellular matrix remodelling, as well as improve hydration in the extracellular matrix [3-5]. The underlying hypothesis is that concurrent delivery may potentiate a synergistic effect, leading to amplified collagen synthesis and enhanced clinical outcomes. Existing literature in regenerative aesthetics increasingly supports the use of multimodal bio-stimulatory strategies, wherein the combined action of compounds such as HA, PDRN, and polynucleotides has been shown to yield superior histological and clinical improvements compared to monotherapy [2-8]. 

To our knowledge, no prior literature has reported the simultaneous delivery of these three bio-stimulatory agents in a single unified bolus, delivered through a closed, sterile Luer-to-Luer system. Traditionally, facial rejuvenation (FR) protocols have involved administering these compounds separately, layered either in distinct anatomical planes or across multiple treatment sessions [2-5]. While effective, this fragmented approach can increase patient discomfort, prolong treatment timelines, raise overall costs, and increase patient fatigue with regard to treatment programmes. Moreover, the disjointed delivery may fail to fully capitalise on the synergistic potential of these agents when used together.

This study emphasises the role of delivery precision. While some practitioners favor microcannula techniques, this protocol uses a 90-degree (perpendicular to skin) injection technique with a 27-gauge needle to place the product accurately in the mid-dermis. This approach provides superior tactile feedback, enabling the injector to gauge depth in millimeters, something cannulas cannot reliably achieve. The result is a more controlled, uniform integration of the product.

The outcomes in this report are promising. All subjects experienced high satisfaction and progressive aesthetic enhancement at six months post-treatment, with no adverse events recorded. This may suggest the protocol can offer a superior efficacy profile compared to conventional multi-step rejuvenation treatments.

Limitations of this case report include the small sample size and the absence of objective histological validation. While subject-reported outcomes and physician-assessed GAIS scores were positive and clinical photography demonstrated marked improvements, larger cohort studies with longer follow-up periods are essential to further validate these findings.

Another limitation is with regard to the use of Profhilo®. While stabilised, non-cross-linked HA products such as Profhilo® have gained popularity for their dual weight hydration and bio-stimulatory profile, their clinical longevity remains limited. Due to the absence of cross-linking, the product is highly susceptible to enzymatic degradation via endogenous hyaluronidase activity, particularly in high-mobility facial areas. Although designed to spread diffusely through the dermis, this same property often results in rapid diffusion away from target structures and inconsistent tissue residency. Furthermore, the lack of mechanical resistance means such products do not form a hydration reservoir with volume retention, limiting their lift and longevity effects to a short post-treatment window. These constraints present a clinical challenge for practitioners seeking longer-lasting dermal support and cumulative bio-stimulation without resorting to traditional volumising fillers. As such, the development of mildly cross-linked HA formulations, particularly those engineered to balance longevity with biocompatibility, represents a promising direction for injectable skin revitalisation technologies. It would be crucial to adopt these mildly cross-linked formulations into future studies to see if they produce a more superior outcome. 

## Conclusions

This case series demonstrates that the aiva® Re-Verse protocol may represent a safe and effective approach to FR. By delivering hyper-dilute CaHA, PDRN, and non-cross-linked HA in a single bolus via a sterile closed transfer system, this technique introduces a novel, multimodal method of stimulating collagen synthesis, improving dermal quality, and restoring facial harmony. In contrast to the widely adopted monotherapy approach, where single agents are administered per session, this protocol leverages synergistic bio-stimulatory pathways for enhanced clinical outcomes. The absence of adverse events highlights the importance of anatomical precision, aseptic technique, and thoughtful compound synergy. Larger, controlled studies with extended follow-up (≥12 months) are warranted to validate these findings and further explore long-term neocollagenesis. Objective modalities, such as high-resolution ultrasound and histological biopsy, may be valuable in quantifying changes in dermal thickness and collagen density. The limitations of non-cross-linked HA in terms of diffusion and degradation suggest that future studies should explore mildly cross-linked alternatives, which may offer improved tissue re-density, hydration retention, and overall clinical outcomes. These preliminary findings position the protocol as a promising integrative tool within regenerative aesthetic medicine, addressing the growing demand for non-surgical facial rejuvenation. 
